# Embracing parenting role in childhood obesity

**DOI:** 10.1186/s12889-023-16039-2

**Published:** 2023-06-12

**Authors:** Jiying Ling, Mekdes Gebremariam

**Affiliations:** 1 Michigan State University College of Nursing, 1355 Bogue Street C241, East Lansing, Michigan, 48824 US; 2 Department of Community Medicine, Global Health, University of Oslo Institute of Health and Society, Oslo, Norway

## Abstract

Active parental engagement is crucial in controlling childhood obesity. However, optimal strategies to engage parents and mechanisms linking parents’ involvement to childhood obesity prevention need further investigation. In this editorial, we provide a background to invite contributions to the *BMC Public Health* collection titled ‘Parenting role in childhood obesity’.

Childhood obesity represents a significant global public health challenge. The prevalence of obesity has doubled in more than 70 countries since 1980; in many other countries it has continued increasing [1]. The World Health Organization’s recent estimates suggest that 1 in 5 children and adolescents are overweight or obese [2]. What is even more concerning is the disproportionate number of children with a lower socioeconomic position that are affected by this pandemic in many settings [3], contributing to health inequities. Adverse impacts of childhood obesity are wide ranging, encompassing social, economic, and health-related consequences. The latter can be both short- and long-term, and include psychosocial, neurological, dental, cardiovascular, respiratory as well as endocrine complications and comorbidities [4]. Body weight is also known to track moderately from childhood into adulthood, making early interventions particularly important.

Obesity at all ages is multifactorial and complex. Multiple models aimed at classifying the potential influences on childhood obesity have been developed. One such model is the social ecological model, a widely used model that recognizes the interplay between factors at the individual (e.g., sociodemographic characteristics, genetic predisposition, knowledge, attitude), interpersonal (e.g., role of family, friends and other social networks), community (e.g., schools, neighborhoods), societal (e.g. cultural norms, media), and public policy levels as they influence health and health behaviors [4]. Interventions aimed at promoting healthy behaviors and preventing obesity should ideally consider this complex interplay of factors. More recently, the need to acknowledge the complexity of the linkages, interactions, and feedback loops among and between these different levels, using a systems approach, has been increasingly promoted [5]. In addition, it has been suggested that adequate participation of stakeholders at different levels (e.g., children themselves, parents in family, schools) and cross-sectoral collaborations are among the factors facilitating the success of interventions in this area.

Parents serve as critical role models to shape children’s healthy lifestyle behaviors including eating behavior, physical activity, sleep, and screen time. Parental poor feeding practices, indulgent parenting style, parental stress, and unsupportive home environment are identified as home and parental characteristics contributing to childhood obesity [6]. The influence of parents on childhood obesity starts from preconception and across the entire childhood to even early adulthood. Current recommendations are calling for aggressive early childhood family-centered obesity interventions [7]. Family is the ecosystem system fostering the growth and development of children while being bounded by social determinants of health (e.g., economic stability, built environment, social context, food accessibility). When developing childhood obesity interventions, family should be the active and core partner for decision-making.

Overall, family-based interventions consisting of training, education, and practices are more effective than children-only interventions, and medium-to-strong intensity of parental involvement results in greater short- and long-term effects on controlling childhood obesity [8]. Active parental involvement is especially significant during maintenance phase to achieve long-term sustained effects. Some evidence even indicates the need to omit children from interventions to be cost-effective, because parents-only interventions have achieved equal or even greater effects on reducing overweight or obesity than targeting both children and parents [9]. Understanding how families as a system organize and manage lifestyle behaviors can help to tailor an intervention to meet family needs.

Although the positive effects of active parental engagement in controlling childhood obesity are established, optimal strategies to engage parents and the additional effects of parents’ active involvement in obesity prevention and intervention are unknown, especially among adolescents. The constant evolving technology (e.g., internet, mobile phone, social media) provides a promising avenue for actively engaging parents particularly hard-to-reach families in intervention research. However, parental participation usually fades over time along with intervention effects, but acceptability of mobile health interventions among parents are high. Moreover, future intervention efforts should focus on assessing parents’ adherence to interventions, examining the beneficial effects on parents’ outcomes, and exploring the associations between parents’ and children’s outcomes. According to the Family System Theory, family members simultaneously affect and are affected by each other, and the overall family dynamics (i.e., family functioning, family cohesion, interpersonal communication) are more powerful than the dynamics between two individual members [10]. Grounded in the Family System Theory, family-based interventions need to consider two types of changes: (1) behavioral changes of members at the family level; and (2) dynamic changes on family structure, rules, communication, and responsiveness.

In summary, parents’ role in the prevention of childhood obesity is critical and widely acknowledged. Prevention of childhood obesity however remains highly challenging with intervention effects that are often modest at best and poorly sustained over time. Adequate prevention efforts would require comprehensive intervention approaches targeting the different levels of the social ecological model. Within such approaches, the active involvement of parents and families remains crucial. More research is in this regard needed to explore the mechanisms through which parental involvement can contribute to positive environmental and behavioral changes. Optimal ways to involve parents in childhood obesity prevention efforts also need to be assessed further.

The aim of this collection is thus to contribute to the complex field of childhood obesity prevention through the publication of studies focusing on the priority areas highlighted in this editorial. We invite authors to submit studies with strong theoretical underpinning and making use of recent advances in statistics to explore causal mechanisms linking parental role and the family environment with childhood obesity. Intervention studies demonstrating how best to actively involve parents in childhood obesity prevention efforts are encouraged. We also welcome studies quantifying the impact of parental/family/home-based interventions on changes in behaviors and body weight, but also assessing mediators of such changes (e.g., family social environment), as there is a lack of obesity intervention studies reporting on the latter changes.

## Data Availability

Not applicable.
